# A Qualitative Exploration of the Process and Experience of Change in Moving on in My Recovery: An Acceptance and Commitment Therapy Based Recovery Group for Substance Use Disorder

**DOI:** 10.3390/bs14121237

**Published:** 2024-12-23

**Authors:** Emma L. Shepley, Mike C. Jackson, Lee M. Hogan

**Affiliations:** 1 North Wales Clinical Psychology Programme, Bangor University, Bangor LL57 2DG, UK; mike.jackson@bangor.ac.uk; 2 Early Intervention in Psychosis, Betsi Cadwaladr University Health Board, Bangor LL57 2PW, UK; 3 Substance Misuse Services, Betsi Cadwaladr University Health Board, Rhyl LL18 3EY, UK

**Keywords:** substance use disorder, acceptance and commitment therapy, group therapy, recovery, behavioural change, qualitative, grounded theory

## Abstract

Moving on in my recovery (MOIMR) is a new, acceptance and commitment therapy (ACT) based group intervention to support recovery from substance use disorder. It was co-developed by, and is co-facilitated with, people in recovery. This study used a grounded theory model to understand the process of change experienced by individuals who completed the group programme. Ten individuals who were abstinent from substances following their participation in MOIMR were interviewed. The model that emerged depicted a chronological series of processes that centred around a core category of gains derived from approaching their emotional vulnerability by leaning in to discomfort (e.g., difficult internal experiences like thoughts, emotions, and physical sensations) whilst pursuing activities that aligned to what mattered to them. Initial key processes indicated that participants experienced a degree of suffering from substance use prior to engagement. Group safety was a key element in fostering connection, normalisation, and cohesion, combined with psychological understanding being significantly derived from those with a lived experience of substance misuse and addiction. Later processes reflecting core ACT mechanisms such as letting go, value-guided action, and acceptance of difficult internal experiences took time to develop; many participants reported completing MOIMR more than once as a means of understanding these components. Limitations, along with implications for clinical practice and future research are discussed.

## 1. Introduction

Substance use disorder (SUD) is characterized as the habitual, compulsive, and continued use of alcohol and/or drugs despite problematic cognitive, behavioural, and/or physiological consequences [1]. SUD is a serious widespread concern; government statistics suggested that close to 300,000 people were in contact with substance misuse services in the UK between 2021 and 2022, a figure which had increased by over 13,000 from the previous year [2]. Common sequelae of SUD include significant distress, impaired functioning, financial crisis, and unemployment [3]. Importantly, SUD is a leading contributor of global disease [4]. The Office for National Statistics [5] showed that SUD-related deaths accounted for 10% of all preventable deaths in the UK in 2018. This was the fourth highest cause of preventable deaths after cancer (35%), diseases of the circulatory system (27%), and the respiratory system (14%).

### 1.1. Psychological Intervention in SUD

SUDs are often chronic in nature and frequently associated with comorbid mental health problems [6]. There is a long history of psychological intervention within SUD, with approaches drawn from a variety of traditions [7] such as contingency management, cognitive behavioural therapy, and motivational interviewing [8]. Reviews of these approaches have suggested that abstinence rates tend to be low and short-lived, with substantial dropout rates [9,10], although contingency management has shown superior outcomes [11]. Furthermore, there is a lack of empirical evidence for approaches that effectively address the comorbidity between SUD and mental health problems [12].

Group-based mutual aid interventions are recommended for promoting recovery from SUD [13]. What exactly constitutes recovery from SUD is hotly debated and agreement on a precise definition is not clear [14]. However, it is generally agreed that it is not sufficient for recovery to be achieved solely by abstinence and it requires improvements across a broad range of biopsychosocial domains [15]. International examples of recovery groups include the Twelve-Step Fellowships: Alcoholics Anonymous and Narcotic Anonymous [16] and SMART recovery [17]. Evidence has suggested that 12-step and non-12-step group outcomes are equivalent, whilst relapse following treatment remains high [18]. The UK government has recommended a wider choice of effective mutual aid groups for the treatment of SUD [19].

### 1.2. Contextual Behavioural Therapy in SUD

With the above points in mind, it is timely to investigate new group-based psychological interventions for SUD. Over the last decade, increasing attention has been given to the application of third-wave and contextual behaviour therapies in the treatment of SUD. These approaches focus on the use of mindfulness and acceptance strategies to reduce the likelihood that internal experiences (such as thoughts and emotions) will lead to substance use [20]. The main difference between contextual behavioural therapies and traditional cognitive behavioural therapies is the emphasis on the context and function of internal experiences, rather than on the content of them [21]. Contextual behaviour therapies, including acceptance and commitment therapy (ACT), have gathered interest over recent years as approaches for effectively addressing comorbidity between SUD and mental health problems; see [22].

ACT has been reviewed in multiple randomised controlled trials, and a recent review of meta-analytic evidence has found it to be an efficacious approach across a broad range of target conditions and has supported the transdiagnostic underpinnings of the approach [23]. Within the ACT framework, substance use is seen as a form of ‘experiential avoidance’ [24], whereby individuals use substances to avoid unwanted thoughts, feelings, and physiological experiences. ACT focusses on approaching these internal experiences with awareness and acceptance, rather than avoidance. Emphasis is also placed upon identifying personal values and supporting individuals to build a meaningful life aligned with these values [25]. The evidence base for the use of ACT in the treatment of SUD is growing: a meta-analysis by Gloster et al. [23] reported a significant small to medium effect favouring ACT over other treatments. Additionally, a systematic review by Byrne et al. [8] and a more recent systematic review and meta-analysis by Krotter et al. [26] found that ACT is useful in treating comorbid SUD and mental health problems.

### 1.3. Moving on in My Recovery

Coproduction has been found to be a vital component of psychological interventions within SUD [27]. Combining coproduction with evidence-based approaches, moving on in my recovery (MOIMR) [28] is a new group-based intervention for SUD. MOIMR draws on ACT to address SUD and mental health problems to support a transition out of substance misuse services. MOIMR was developed following consultation with people with lived experience of recovery and professionals working in the area to identify what was most helpful in the recovery process. It aims to bridge the gap between formal treatment provision and mutual aid. Sessions are co-facilitated by professionals and graduates of the programme. The programme is aimed at individuals who have achieved abstinence and are considering a transition out of treatment services.

MOIMR consists of twelve weekly, two-hour sessions. Topics considered include supporting wellbeing, dealing with anxiety and low mood, building/rebuilding relationships, planning for relapse, working with loss, self-identity, and stigma, all of which are delivered from an ACT-based perspective. Each session begins with a ‘check-in’, where group members discuss how they have applied MOIMR skills throughout the past week, and ends with a ‘check-out’ where group members agree on how they will apply learning throughout the next week by setting a challenge. The check-in and check-out also give group members the opportunity to share experiences and to offer support to others. The challenges that are set each week encourage commitment and action towards values. An initial pilot study demonstrated promising results including improved social functioning, reduced low mood and anxiety, and decreased experiential avoidance; improvements that were maintained at a three-month follow-up [28]. Plans are underway for a larger randomised control trial.

Research of psychological interventions in SUD (including ACT-based interventions) has largely focussed on quantitative measures [12,29]. However, it is recognized that this type of research does not uncover the deeper dimensions involved in the process of recovery and fails to explore mechanisms for change [14]. It has been proposed that future research should place a larger focus on understanding the change processes involved within recovery [30]. Thus, when evaluating therapeutic approaches it is important to investigate not only if an approach works, but also how it works for individuals.

A literature review found only one published study employing qualitative methodology to explore the application of ACT within an SUD population [12]. No studies were found which pertained to the processes of change during an ACT-based intervention for SUD. Grounded theory methodology [31] was selected for the present study due to the emphasis this approach places upon developing a model grounded in the experiences of participants. Grounded theory is appropriate when little is known about a phenomenon, and facilitates uncovering of the processes inherent to the area of inquiry [32], such as the process of change through engagement with a recovery group. Grounded theory has been applied to understanding the processes involved in therapeutic groups including mindfulness-based approaches for chronic pain [33] and distressing voice hearing [34]. The aim of the present study was to use grounded theory to develop a model of the change processes that occurred through engagement in MOIMR, derived from the experiences of participants who had made steps towards recovery.

## 2. Method

### 2.1. Design

Grounded theory methodology, following the procedures outlined by Strauss and Corbin [31], was used to generate and analyse data.

### 2.2. Participants

Participants were recruited via NHS substance misuse service staff within Betsi Cadwaladr University Health Board in Wales, UK. These staff members provided information about the study to all of those who met the recruitment inclusion criteria.

#### 2.2.1. Inclusion and Exclusion Criteria

Participants met the inclusion criteria if they had completed MOIMR within the past 12 months and had attended a minimum of 9 out of the 12 group sessions. In order to take part, participants must have been in recovery and abstinent from substances for a minimum of three months after completing MOIMR.

#### 2.2.2. Participant Characteristics

Ten participants took part in the research and none of these participants dropped out of the study. Within the pragmatic constraints of the project and sample, recruitment ceased at ten participants as by this point there were no further significant developments to the emerging theory. The sample comprised two female participants and eight male participants. All participants were originally from Britain and of white, Caucasian ethnicity. The mean age was 52.9 years (*SD* = 7.0), with ages ranging from 36 to 64 years. Participants were previously addicted to alcohol (n = 4), heroin (n = 2), crack cocaine (n = 1), amphetamines (n = 1) or multiple substances (n = 2). All participants reported long histories of addiction ranging from 15 to 35 years. The length of time in recovery at the point of interview ranged from 6 months to 2 years.

#### 2.2.3. Ethical Considerations

Prior to agreeing to take part, all participants were provided with an information leaflet with details about the research. This leaflet clearly outlined how the data they provided would be confidentially treated. Informed consent was obtained from all participants. Participants were informed that participation was voluntary and they were free to withdraw at any point without their treatment within substance misuse services being impacted; no participants chose to withdraw. The capacity to give consent was assessed by asking participants to talk through their understanding of the benefits and potential risks of taking part in the research. The first author, who completed the interviews and analysis, was not involved in any aspect of the participants’ care within substance misuse services.

### 2.3. Procedure

NHS substance misuse staff approached potential participants for the study and offered them an Information Sheet. Interested participants were asked to complete and return a form to state they were interested in taking part and were willing to be contacted by the researcher. The first author contacted participants over the telephone, answered questions about the research, and arranged to carry out face-to-face interviews.

A semi-structured interview schedule was used to gather data (see Supplementary Materials, S1: Interview Schedule). The schedule was drawn up by the research group; as the aims of the study were to examine change processes and turning points, the questions centred on these topics. Feedback was sought regarding the appropriateness of the questions from a MOIMR graduate and group facilitator prior to the commencement of the study. In line with grounded theory methodology, the interview schedule evolved throughout the data collection process. General topics included life before MOIMR, the experience of the group process, what learning was acquired from MOIMR, and what aspects were helpful and unhelpful. The interviews were conducted in a participant-centred manner, with open questions and follow-up questions and prompts where appropriate.

Ten interviews were conducted over four months between November 2019 and February 2020. All interviews took place within a clinical setting. Each interview was recorded using an encrypted dictaphone and transcribed by the first author with identifying information removed. The interviews were carried out in three stages. During the first stage, open questions were asked to allow initial ideas to emerge; examples included “How has MOIMR played a role in the recent changes you have made in your life?” and “Could I ask you to describe the most important things you learnt through MOIMR?”. In keeping with theoretical sampling, ref. [31] the interview schedule was revised after each interview and evolved throughout the data collection process as categories and subcategories began to become clear; thus, during the second stage, questions were more focussed around the emerging categories (e.g., “Which skills or tools did you find most helpful?”, which was focussed around the emerging category of ‘action’ and “Can you tell me about something difficult that has happened in your life recently, and how you applied MOIMR principles to cope with it?”, which was focussed around the emerging subcategory of ‘understanding the impact of maladaptive strategies’). The final stage was used to confirm hypotheses and strengthen the validity of the model. Participants were asked questions such as, “Were there some elements of MOIMR learning that longer took to understand and apply than others?”

The first author maintained a warm, engaged, and open approach when conducting the interviews. Only the author and the participant were present during all interviews. Plenty of time was given for participants to formulate their responses to questions and the author checked in with each participant at the end as to whether they felt the interview had been a true reflection of their experiences and if they had anything more to add. All participants were keen to share their stories and gave significant detail about their experiences. Therefore, the author did not feel it necessary to go back to participants for further clarification.

### 2.4. Data Analysis

All analysis was carried out by hand by the first author (see Supplementary Materials, S2: Worked example of analysis and model development process). In keeping with the grounded theory method, analysis was carried out throughout the study and was used to shape the data collection process. Open coding, using line-by-line analysis, was used initially to generate concepts. These concepts were transferred to cards, which were sorted and grouped into higher-order categories as the analysis took shape. Properties and dimensions of the categories were identified, and axial coding was used to clarify relationships between the categories. As the core category emerged, selective coding was employed, which involved revisiting the categories and delineating their relation to the core category. Constant comparison and memoing were used throughout to ask whether a concept had been seen before to note ideas and reflections and to use diagrams to support the development of the model.

#### 2.4.1. Quality Assurance Methods

The first author made use of theoretical memoing to note preconceptions about the data and any emotional reactions to the data generated, with the intention of maintaining awareness of her perspective. Additionally, a coded transcript was audited by the second and third authors to ensure credibility within the coding and category generation.

#### 2.4.2. Author Reflexive Statement

The author is a white, female trainee clinical psychologist with no personal experience of SUD or substance misuse services. Additionally, she had no professional experience in delivering groups to support recovery from SUD. That said, the author had professional experience in using ACT to support individuals presenting with various mental health problems. She holds the belief that ACT is an effective and helpful therapeutic approach.

The author was led by the data during the analysis. However, her lack of personal and professional experience with SUD could potentially have limited her ability to fully identify concepts related to this. In order to account for this, the third author, who is a clinical psychologist with extensive experience working with individuals with SUD, oversaw the analysis process. It is also worth noting that the third author was responsible for the development of MOIMR. Therefore, it is acknowledged that the emerging categories may have been influenced by the author’s professional experience of and belief in the benefits of ACT and the third author’s vested interest in MOIMR as an effective intervention. Efforts were made to account for this by intentionally searching for negative incidences, sorting codes according to those that were and were not in line with ACT, and actively encouraging participants to discuss any unhelpful aspects of MOIMR. The first author’s independence from MOIMR made it easier for participants to discuss negative perspectives. Additionally, the second author was not involved with MOIMR and had less experience of using ACT. Therefore, the second author supported the first and third authors to recognise and look for biases during the analysis process.

## 3. Results

### 3.1. Interviews

The average length of interviews was M = 48 min (*SD =* 15.0), with interviews lasting between 20 and 80 min. The mean amount of data collected from the interviews was 7092 words (*SD =* 1883.5), with interview transcripts ranging from 3142 to 10,676 words.

### 3.2. Overview of the Model

**Figure 1:**
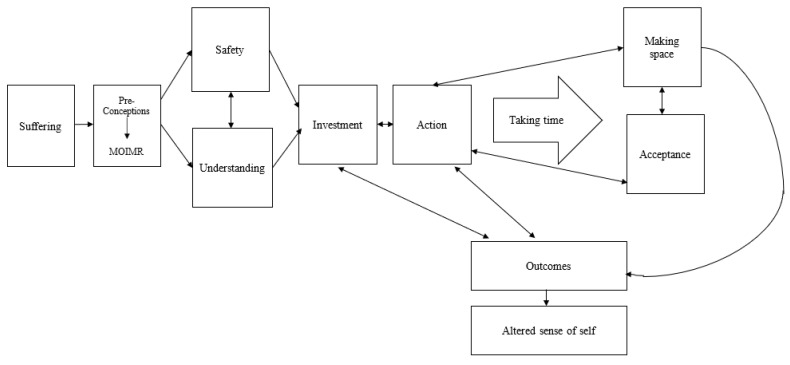
Model of the process of change through MOIMR.

Figure 1 is a model of the process of change made throughout MOIMR. Categories and subcategories are represented in Table 1 and highlighted in the text in bold. The model depicts the change processes that occurred through participants’ engagement with MOIMR, grounded in the descriptions of participants’ experiences. The model begins prior to MOIMR with an experience of suffering as a result of substance use, timing factors, and reaching a turning point of deciding to make changes. Positive preconceptions supported initial engagement with MOIMR. The first stages of the group experienced by participants were feeling safe in the group setting and then beginning to understand substance use from a psychological perspective, which were necessary for further changes to be made. Investment into MOIMR and recovery followed. A focus of MOIMR was to identify what is important in life (values) and support participants to take behavioural action that was consistent with this. Positive outcomes followed these stages, which had a bi-directional relationship with the processes, strengthening their continuation. Time was taken to reach the final stages of acceptance of emotions and making space for difficulty, which often involved completing MOIMR more than once.

### 3.3. Core Category

The core category that emerged from the analysis and could be seen to some degree within all other categories, were the gains of engaging in MOIMR and recovery consistent behaviours. Given the group nature of MOIMR, these gains involved a strong social component.

For all participants, there was a great degree of suffering which had resulted from substance use prior to engaging with MOIMR. Thus, the reinforcement that was gained from engaging with recovery-consistent behaviours through MOIMR in terms of social connection and improvements in daily life was sufficient to support participants to overcome barriers including fear of being in a group setting, motivation, and the difficulty of getting in touch with some painful experiences. Engaging in recovery-consistent behaviours began to hold an increasingly greater degree of reward than engaging in substance use.

### 3.4. Category A: Suffering

This category represents the large degree of suffering that participants experienced prior to engaging with substance misuse services and prior to MOIMR. Participants explained that there had been several **detrimental impacts** resulting from their substance use, including within their families, relationships, and work, for example:

P4: “*I wasn’t seeing my daughter I was drinking more and more and more and then I lost me job as well… and it went downhill from there really…*”

Participants described **feeling stuck**, with a lack of meaning and a general dissatisfaction with life. This resulted in **seeking help** from substance misuse services; however, many participants described that they had been with these services for many years and had not made any significant changes in their substance use. Various **timing factors** were described by participants prior to MOIMR; physical and/or mental health problems were often mentioned. For example:

P5: “*I felt so low and got to such a low place… I was both physically and mentally drained… literally drained… and you know I felt there was only one way I could only go up here cos you know that was really how bad it was*”

Due to the culmination of these factors, all participants had a **turning point** story, which contained decisions made to take steps towards recovery. For all participants, this occurred prior to MOIMR:

P7: “*it was my time you know to turn it around… I just couldn’t carry on like that… I’d had enough you know; it wasn’t just the drugs it was all the crap that went with it. I couldn’t do it anymore; mentally I felt like I was gonna have a nervous breakdown… I was on the edge*”

Many participants described **positive pre-conceptions** of MOIMR which they had developed through hearing of the benefits others had experienced. Others described positive experiences of other ACT-based recovery groups they had taken part in prior to MOIMR. These had led participants to want to take part in MOIMR.

P4: “*I’d seen the changes that some of the other lads made, and I thought yeah alright I’ll give it a try*”

### 3.5. Category B: Safety

This category represents the first process that occurred when participants began MOIMR. Participants described that entering a social setting, after what had often been an extended period of isolation due to substance use, was **scary at first.**

P6: “*yeah anxiety ridden… I was really bad. I didn’t live in the toilet but I spent a tiny bit longer than I should have done there… I’d been out of the world for so long and then coming back it was a real shock*”

This was alleviated through the creation of a non-judgemental environment; participants described the feeling that **“We were all in the same boat”.** This process was enabled via warmth, genuineness, and openness on the part of the group facilitators. Group members began to feel safe to share experiences, of which they may have felt highly ashamed and kept hidden for many years. Hearing that others in the group had had similar experiences was de-stigmatising and normalising:

P3: “*it was good… open friendly people and it was very warm and welcoming… it was nice because people have been in your situation you know and you can tell that about the group… whereas you get a lot of judgemental people you know stigma and that… there’s no feeling of that, no stigma or anything; it’s just normal people who’ve had a bad time*”

P8: “*you speak about things and you think ‘I was the only one who’s been there’, sort of thing and it sort of opens doors and you think ‘Christ we’re all in the same boat here’… it is good to hear I’m not the only one… I’m not a freak*”

Participants described a strong **connection** with other group members; many participants formed strong bonds and friendships. These positive relationships were important as participants often described having to separate themselves from existing friends and relationships which may have perpetuated substance use:

P8: “*at the start of the programme I was quite nervous thinking ‘oh do I want to talk about that’, ‘should I mention this?’ but eventually I’d open up… and being able to open up and offload that in front of people who understood that helped in a big way*”

P1: “*I made two very good friends out of it you know and that’s been missing… that’s been missing in my life… I hadn’t made new friends for 20 years while I was on the drink*”

The connection evolved into a sense of a **shared journey towards recovery**:

P6: “*I think there’s this thing with being in a group… even if it’s not spoken it’s an incentive purpose*”

The group employed a flattened hierarchy approach where the **facilitation style** involved a degree of self-disclosure and participation in weekly challenges etc. This contributed to the overall feeling of safety.

P5: “*there was no us and them… we were all in it together, helping each other out*”

### 3.6. Category C: Understanding

Participants described beginning to develop an understanding of their substance use; all explained that the content of the programme was extremely **resonant** with their own experiences:

P8: “*it was almost as though I wrote the bleeding thing… thinking oh he’s [facilitator] got it all on paper what I’ve got in here [points to own head]*”

The material was described as accessible, using everyday language to explain ACT ideas and psychoeducation. Participants explained that having this understanding meant they felt able to begin to take steps to change their addictive behaviours:

P7: “*it **puts in layman’s terms** what’s going on mentally you know*”

P7: “*to understand what was going on and take it instead of getting all these emotions and not knowing, like I had done before… to be able to break it down and understand and how to deal with cravings as well…*”

Participants reported they appreciated the **balanced structure** which allowed opportunity for openness and sharing as well as covering structured topics each week. For example:

P3: “*there’s like a topic in them [group sessions] and stuff; it was like keeping my mind awake and focussed… I’ve been to other groups and that’s more like people just go there to have a chat really*”

### 3.7. Category D: Investment

Some participants described an initial lack of **motivation** to attend the group and a lack of or only partial investment in recovery. A sense of scepticism around the benefits of MOIMR was described, along with a reluctance to engage in group discussions and complete tasks set within the group:

P6: “*’should I go home?’, ‘should I not?’ …you know I just didn’t really wanna engage*”

Participants described that motivation began to build through **hearing the stories of others** who had recovered through the group and through the peer facilitators:

P2: “*seeing the positivity of those people who’d done it, I’d say in my case, that gave me the will to do it*”

Participants described beginning to tentatively try things out and beginning to **see the benefits:**

P4: “*I sort of did start applying things and you know like more motivation came and I was, like, doing things more*”

Motivation continued to grow throughout the group, comparable to a **snowballing effect**:

P1: “*[we] set a challenge each week… for the first few weeks I never did one… but by the end of it I was doing every one*”

Participants described that they began to develop motivation to make **changes in other areas of their lives**, outside of their recovery:

P7: “*it starts definitely with MOIMR like coming here volunteering…it led to all of this for me you know… I think if it weren’t for that I’d probably have started using again*”

### 3.8. Category E: Action

This category refers to participants taking action to implement changes in their lives through changes in their behaviour. Participants described a lack of **meaning** prior to MOIMR:

P1: “*empty I suppose is the word that strikes to mind… there was just nothing in my life no structure*”

Weekly attendance at MOIMR provided a **focus point** in the lives of participants. Engaging with the weekly challenges was described as giving participants a way of spending their time which felt productive, and supported participants to begin to implement structure into their lives.

P2: “*it made me realise I had other things I wanted to do… I’d been through a phase of purposeless like I’ve got nothing to do, well there’s no point doing that…*”

P6: “*It’s good cos in the chaos of the addiction I just lurched from one situation to another and everything was so up in the air*”

The idea of **considering one’s values and using these to guide action** was emphasised in MOIMR with the metaphor of “anchor points”; for most participants, this meant using activities which were related to their values to guide them when difficulty arose. This gave participants a way of considering alternatives to their substance use which connected them with what was important to them.

P1: “*If I start feeling down with the depression I don’t think now ‘right I’m gonna go and get a drink’… what I do now is use me anchor points which is music, fishing, decorating… I’ve got quite a few anchor points… reading books… the things I never, ever did before I went to the group*”

Participants described a process of placing **priority in recovery** and building a substance-free life. This was a difficult process; for many participants, it meant ending relationships and friendships that perpetuated addictive behaviours.

P7: “*my partner did still use so I just always got dragged back in to it… so I had to get rid of him to stop, so that was a big thing you know… cos we were together 20 years*”

### 3.9. Category F: Taking Time

Several participants had **completed MOIMR more than once** and emphasised that without this they would have struggled to make considerable changes:

P6: “*if anything, I’d say it doesn’t all sink it at once… it took me doing it twice to really absorb it*”

Those who had only completed the group once discussed wanting to do it again, suggested they felt the group should be longer or that there should be a follow-on group. Some participants explained that some of the processes (see Categories H & I) took a long time to grasp and explained that without completing the group more than once they would have struggled to apply them.

### 3.10. Category G: Moving from a Place of Avoidance to Making Space for Difficulty and Discomfort

Participants described a long history of substance use as a means of avoiding uncomfortable emotions and a lifestyle which involved avoidance of numerous situations:

P1: “*I just hid behind the bottle*”

MOIMR supported participants to **understand the consequences of maladaptive coping styles** (typically using substances to manage uncomfortable thoughts or emotions):

P7: “*I’d of just wanted a hit I wouldn’t have even… even just simple things like paying bills… learning if there’s a problem dealing with it straight away cos if you bury your head in the sand they just get worse*”

The term **“lean in”** is used within MOIMR which refers to moving towards, rather than avoiding difficulties that arise. This was typically used to refer to internal experiences including thoughts and memories, and situations that arose in life. Although all participants described some aspect of this, this idea was more apparent in the descriptions of participants who had completed MOIMR more than once. For example, this quote is from a participant who had completed MOIMR three times:

P4: “*you sort of turn to that fear and just like do it… it’s been really hard but now I see the benefits are brilliant*”

Participants were able to describe how they had applied this to their day-to-day lives, for example, one participant described a recent experience of losing a loved one:

P8: “*I think the whole family was expecting me to crash you know, to go back down the bad line but what I did instead of that I sort of threw myself in to everything. I organised the burial, the funeral, everything… I knew through the programme like dwelling on things and just thinking ‘well one bag will switch it off for today’, it’s not gonna stop it’s gonna carry on; I knew that*”

Participants also described learning to **make space for emotions**, both pleasant and unpleasant, and the idea of a valued life being one that involves the experience of some unpleasant emotions. This was also apparent to a greater degree within the descriptions of participants who had completed MOIMR more than once.

P9: “*I’m gonna get peaks and troughs as you do that’s life not an excuse to use... that’s how it was before you know*”

Participants described beginning to acknowledge thoughts rather than pushing them away:

P10: “*I think I’d learned to lock them away [thoughts] and, yeah, by doing that they’re just gonna keep coming back and you’ll have to deal with it*”

MOIMR supported participants to **get in touch with losses**. Loss was very evident in the lives of participants, for whom their addictive behaviours may have been a means of blocking out the pain associated with loss. Loss was also a common consequence of substance use. For others, loss was relevant to the drugs themselves. All participants explained that through MOIMR they got in touch with their losses, and all explained that this was the most difficult part of the group and their overall experience of recovery.

P7: “*facing that loss without drugs, learning that cos you’re sedated for so long all your emotions are sedated as well… so facing that, having to think about that instead of just pushing it to the back of my mind… that was the hardest thing*”

**Mindfulness strategies** were described by some participants as a means of allowing space for and coping with the intensity of emotions:

P7: “*If I have a craving, it’s ok don’t panic…I breathe do my mindfulness… it helped me not to react to my emotions mindfulness did*”

Although mindfulness concepts are relevant to many of the processes (e.g., the mindfulness concept of observing thoughts), using mindfulness as an explicit strategy was not discussed at all by some participants, suggesting that it was not something that everyone took on board.

### 3.11. Category H: Acceptance

This category reflects another process which was more apparent within the descriptions of participants who had completed MOIMR more than once. The idea of **“letting go”** is emphasised within MOIMR. For all participants, this referred to trying to let go of regrets about their past behaviour and their past selves, such as years spent with little focus in life other than seeking substances, or the impact of substance use on relationships. Some participants described fully applying this idea, whereas some were clear that there were some things they did not feel they could let go of.

P8: “*I’ve carried a rucksack full of problems my whole life… I try to let it go now, what’s done is done*”

P10: “*I’ve let go of feelings about my addiction… there’s still a few things that I have a lot of problems about letting go, mainly with family*”

These contrasting responses indicate for some, there was an element of remaining struggle with their past selves and past behaviour towards others.

Some participants described the process of learning to gain **distance from thoughts.** This involved the application of mindfulness principles and learning to take an “*observer*” perspective on one’s thoughts in relation to addiction. For example:

P7: “*before I was like no, no, no don’t, don’t, don’t… and you’re mentally at war with yourself… but understanding, ok, I’m just craving… accepting that I’m craving and then saying ‘no thank you… I’m not doing it today’*”

This captures the process of moving from a place of being “at war” with one’s mind to being “at peace”.

### 3.12. Category I: Outcomes

**Integration** of MOIMR into the lives of participants was facilitated via the check-in process at the start of each group. Participants explained that hearing from others about how they had applied the group principles helped them to think about how they could apply it to their own lives:

P2: “*I picked up on it so much more because other people had brought it up in their check in… so it’s reiterating an important point of the course, so that checking in process is great*”

All participants described a gradual process of integrating learning from MOIMR, which led to positive outcomes. These positive outcomes supported participants to continue to apply MOIMR principles

Participants described feeling **de-stigmatised** and having an increased sense of self-worth

P8: “*I don’t feel as little anymore… as much of a waste of space… more of an equal, you know*”

A sense of **achievement** from the acknowledgement of their success in recovery by self and others was described:

P7: “*it’s little steps and every time I feel proud and a little rewarded*”

A number of participants found that by engaging with others in the group, by carrying out weekly challenges, and by moving towards difficulty gave an increased sense of **confidence:**

P5: “*I’ve been very much more confident; I can take things in my stride without panicking, ‘oh what am I going to do?’ ‘I must have a drink’*”

Through mentalising, being able to see others’ perspectives, and without the strain that substance use placed on relationships, several participants spoke about **improvements in their relationships with others.**

Participants described an overall **enrichment** of their lives:

P9: “*I’ve learnt to go and appreciate the outdoor walks and mountains… go swimming with my son. I enjoy things like that now they’re not a chore I love doing them… I’m in touch with that side of me now*”

All participants spoke about wanting to **altruistically** use their own experience of recovery to help others. Most participants described wanting to stay involved with MOIMR groups as facilitators and many also described aspirations towards careers in recovery work:

P3: “*That’s what I’m perusing now… going into you know helping people… caring and stuff, maybe… becoming like a keyworker myself or helping people*”

This final subcategory reflected an investment in the identity as someone in recovery.

### 3.13. Category J: Altered Sense of Self

This category refers to the process of altered perspectives participants held about themselves and their lives, which was apparent as a later process in all participants’ experiences of MOIMR and recovery in general.

Participants spoke about beginning to **open to the possibility of a different life** outside of one dominated by substance use:

P1: “*It just made you realise that alcohol wasn’t your life whereas before it was; I was always thinking ‘right where’s the next drink coming from?’… before the group I didn’t like me, I didn’t like the world… and now, I’m slowly but surely getting to like myself again*”

MOIMR addresses the ideas of both stigma and **self-stigma**. Participants described realising that a lot of the perceived stigma they felt was internally generated and that their assumptions of how they were viewed by others were not necessarily accurate:

P7: “*you don’t know what other people are thinking, you know, you’re just presuming cos you’re judging people by your own standards; so you just put yourself down mentally so you think everyone else is thinking the same, but they don’t…*”

P8: “*I’ve always had this fear that it’s written all over me you know, druggie, and all this sort of thing but it wasn’t so*”

Finally, a sense of **new self** was reflected in the way participants spoke about their past selves and their past addictive behaviour:

P9: “*I’ve reinvented myself in a way… regrown… cos I never got chance, did I, ‘cause I was always consumed by drugs*”

## 4. Discussion

This study is the first attempt to build a qualitative model of the process of change from an ACT-based therapy group to support recovery from SUD. The study’s findings are considered in the context of the existing literature and established theory. Clinical implications, suggestions for future research, and the limitations of the study are also outlined.

Principally, MOIMR can be conceptualised as a group programme that is underpinned by an ACT-based model that increases participants’ willingness to experience their emotional vulnerability. Unlike, traditional cognitive therapy, which seeks to modify the content of thoughts and beliefs [35], MOIMR encourages participants to move towards their difficult thoughts, emotions, and physical sensations, to make space for them, and to open up to these internal experiences without seeking to change them, using skills such as leaning in. It also encourages diffusion from internal experiences, such as thoughts and memories, using skills such as letting go. What is clear from the experiences of the group participants is that prior to attending MOIMR they had worked hard to avoid leaning in: in fact, they had used substances and employed other behaviours to move away from discomfort for many years and the consequences resulted in a high degree of suffering, self-blame, and shame.

Participants were able to identify two mechanisms and benefits of leaning in towards difficult experiences. First, in moving away (i.e., through excessive substance use) despite the short-term benefit of experiential avoidance, they experienced a high degree of suffering in the long term. The detrimental long-term impact of substance use had increased to the point where this form of experiential avoidance was no longer effective. This is consistent with reinforcement theories of addiction (for a recent review see Wise & Koob [36]) and with the ACT-based explanation of addiction [24]. Leaning in naturally reduced the negative consequences of damaging maladaptive coping strategies.

A second benefit of leaning in was that it enabled participants to connect to those things that really mattered to them (e.g., organising a funeral despite the discomfort of experiencing a loss). Of note, leaning in to emotional vulnerability is not an attempt to eradicate discomfort, as one moves towards those things that matter then feelings of discomfort increase rather than decrease (e.g., thoughts of failing and feelings of doubt can become more prominent). In choosing to lean in and in making space for discomfort, participants were able to derive reinforcement from many other areas of their lives (e.g., in helping others), which resulted in feelings of pride, reward, and confidence.

Participants learned the MOIMR skills through their own direct experience and vicariously by observing the change and positive outcomes shared by their peers. In fact, listening to the experiences of change from others in recovery (often those with more recovery time or from those who had completed the programme previously) was deemed highly beneficial. The importance of peer role models such as these has been recognised in the literature and identified in other qualitative accounts of recovery [37].

The safety within the group facilitated skill attainment, although many only felt ready to embark on the MOIMR style of recovery after experiencing the group for the second time. The practical steps delivered in “*layman’s terms*”—of what was a new/novel concept—described by those with lived experience supported the process of change. There was a recognition that change occurred through the doing: it required investment and action; again, the experience of a shared journey, modelling by facilitators, and the commitment to challenges were crucial to the change process. A process of change from initial scepticism and ambivalence, followed by increased commitment, and then taking action towards change is consistent with the contemplation—preparation—action process depicted by Prochaska and DiClemente’s Stages of Change Model [38].

The social support, connection, and identification that resulted from engaging with MOIMR was highly positively reinforcing, and thus supported the continuation of recovery-consistent behaviours and was sufficient to support participants to overcome barriers. Entering a group setting after often long periods of social isolation was an intimidating experience for participants. The non-judgemental and warm atmosphere of the group setting was essential in alleviating this initial discomfort. Participants began to feel safe to share experiences and described that hearing similar experiences from others in the group was extremely normalising and de-stigmatising. This supported an ongoing sense of connection and identification with the group and led to the development of a shared purpose within the group in terms of a movement towards recovery. Elements of this category are related to social support, which is a well-established component of successful recovery groups [39].

Participants’ descriptions of a sense of belonging and shared purpose were consistent with the components of a psychological sense of community [40]. These findings were in line with previous qualitative studies, such as DeLucia, Bergman, Formoso, and Weinberg [41] who investigated the successful components of recovery groups from a 12-step perspective. The altered sense of self-described by participants (e.g., “*I’ve reinvented myself in a way*”) was a position that offered new insights and opportunities. The MOIMR facilitated insights that were transformative (see Miller & C’de Baca, [42]).

### 4.1. Clinical Recommendations

Together with the findings of the pilot study [28], the current study supports the benefits of MOIMR as an intervention to promote recovery from SUD. Participants suggested that the group should have a follow-on group.

The structure of MOIMR, particularly the check-in was especially helpful and appeared to be key to several of the categories, including group cohesion, increased the sense of safety, understanding, and application of group learning into daily life. Weekly challenges were also important to support the application of group learning and strengthen commitment. The coproduced element of MOIMR was reflected in participants’ accounts. Consulting with those with experience of recovery from SUD in the development of MOIMR meant that the group closely resonated with the participants’ own experiences. Additionally, hearing from peer facilitators who had graduated from MOIMR, and who had made considerable changes as a result, was extremely helpful. These points hold implications for the development and delivery of psychological group therapy in general, both within substance misuse services and across mental health services more widely.

### 4.2. Limitations and Suggestions for Future Research

An important limitation of the current study was the focus on participants who had benefited from MOIMR. Although this allowed a greater focus on change processes towards recovery, it limited the model’s ability to account for those who did not achieve change. This focus was pragmatic given the time and resource constraints of the research. It is acknowledged that this meant only partial saturation was met and the constant comparison method could not be fully applied. It would be beneficial for future research to test the validity of and develop the current model by recruiting participants who did not benefit from, or who dropped out of MOIMR. It would be helpful to examine whether variation in the categories of the current model can account for both change and lack of change. Additionally, it would be helpful for future research to consider including substance misuse service staff with experience in delivering MOIMR to examine whether the model is consistent with their observations of change processes within service users. Further sampling in this way would allow the research to reach full saturation.

Input was sought from a graduate of MOIMR in the design of the study and development of the interview schedule. However, it may have been helpful to draw further on this input during the coding process, given the author’s lack of personal experience of addiction. Lastly, the current study recruited a relatively small, entirely Caucasian and majority male sample of low to middle socioeconomic status. This is fairly representative of the population who typically access substance misuse services in North Wales, UK, where the majority of MOIMR groups are offered; however, this sample limits the explanatory power of the model. If MOIMR were to become available more widely, it would be beneficial to test the model within more diverse samples.

## 5. Conclusions

Overall, this study adds to our understanding of recovery from SUD and how ACT can support this process. Using grounded theory methodology, a model was developed to explain the process of change through an ACT-based recovery group. The model depicts the core processes involved in this change, which follow a chronological order, centred around a core category of approaching one’s emotional vulnerability.

## Figures and Tables

**Table 1:** Categories and Subcategories.

Categories	Subcategories
A. Suffering	Detrimental impacts of addictive behaviours
	Feeling stuck
	Seeking help
	Timing factors
	Pre-conceptions
B. Safety	Scary at first
	“We were all in the same boat”
	Connection
	Shared journey
	Facilitation style
C. Understanding	Resonant
	“Puts in layman’s terms”
	Balanced structure
D. Investment	Motivation
	Hearing the stories of others
	Seeing the benefits
	Snowballing effect
	Making changes in other areas
E. Action	Meaning
	Focus point
	Value-guided action
	Placing priority in recovery
F. Taking time	Completing MOIMR more than once
G. Making space for difficulty and discomfort	Understanding of maladaptive coping strategies
	“Leaning In”
	Making space for emotions
	Getting in touch with loss
	Mindfulness
H. Acceptance	“Letting Go”
	Gaining distance from thoughts
I. Outcomes	Integration
	De-stigmatised
	Achievement
	Confidence
	Improvements in relationships
	Enrichment
	Altruism
J. Altered sense of self	Opening to the possibility of a different life
	Reduced self-stigma
	New self

## Data Availability

The data presented in this study are available on request from the corresponding author due to participant privacy restrictions.

## Supplementary Materials

The following supporting information can be downloaded at: https://www.mdpi.com/article/10.3390/bs14121237/s1, S1: Interview Schedule; S2: Worked example of analysis and model development process.
