# Supplementary material for: A Qualitative Exploration of the Process and Experience of Change in Moving on in My Recovery: An Acceptance and Commitment Therapy Based Recovery Group for Substance Use Disorder
Source: Behav Sci (Basel). 2024 Dec 23;14(12):1237. doi: 10.3390/bs14121237 (PMC11673865; doi:10.3390/bs14121237)
Supplement: Supplementary file 1 [file behavsci-14-01237-s001.zip › behavsci-3274256-supplementary S1.pdf]

Age:

Gender:

Substance/s previous issues with:

Questions about the experience and process of moving towards recovery:

1. Could you describe your life before attending MOIMR

2. Could you tell me about the person you are now?
    a. What positive changes have occurred in your life since MOIMR?
    b. Could you tell me about how your coping strategies have changed since attending MOIMR?
    c. Tell me about the strengths that you discovered and developed through attending MOIMR.

Questions about MOIMR groups:

1. Could you tell me what made you decide to take part in MOIMR?

2. Could I ask you to describe the most important things you have learnt through attending MOIMR?

    a. Are there any particular sessions or moments that stand out in your mind?

3. Which parts of MOIMR did you find most helpful?

4. What has been unhelpful/ what could have been different about MOIMR?

Questions about the future/ maintaining recovery:

1. What do you think are the most important ways to maintain recovery?
2. Where do you see yourself in two years? Describe the person you hope to be then.

Final questions:

1. Is there anything you might not have thought about before that occurred to you during this interview?
2. Is there something else you think I should know to better understand how MOIMR helps people to change?
