# Supplementary material for: A Qualitative Exploration of the Process and Experience of Change in Moving on in My Recovery: An Acceptance and Commitment Therapy Based Recovery Group for Substance Use Disorder
Source: Behav Sci (Basel). 2024 Dec 23;14(12):1237. doi: 10.3390/bs14121237 (PMC11673865; doi:10.3390/bs14121237)
Supplement: Supplementary file 1 [file behavsci-14-01237-s001.zip › behavsci-3274256-supplementary S2.pdf]

Excerpt of a coded transcript:

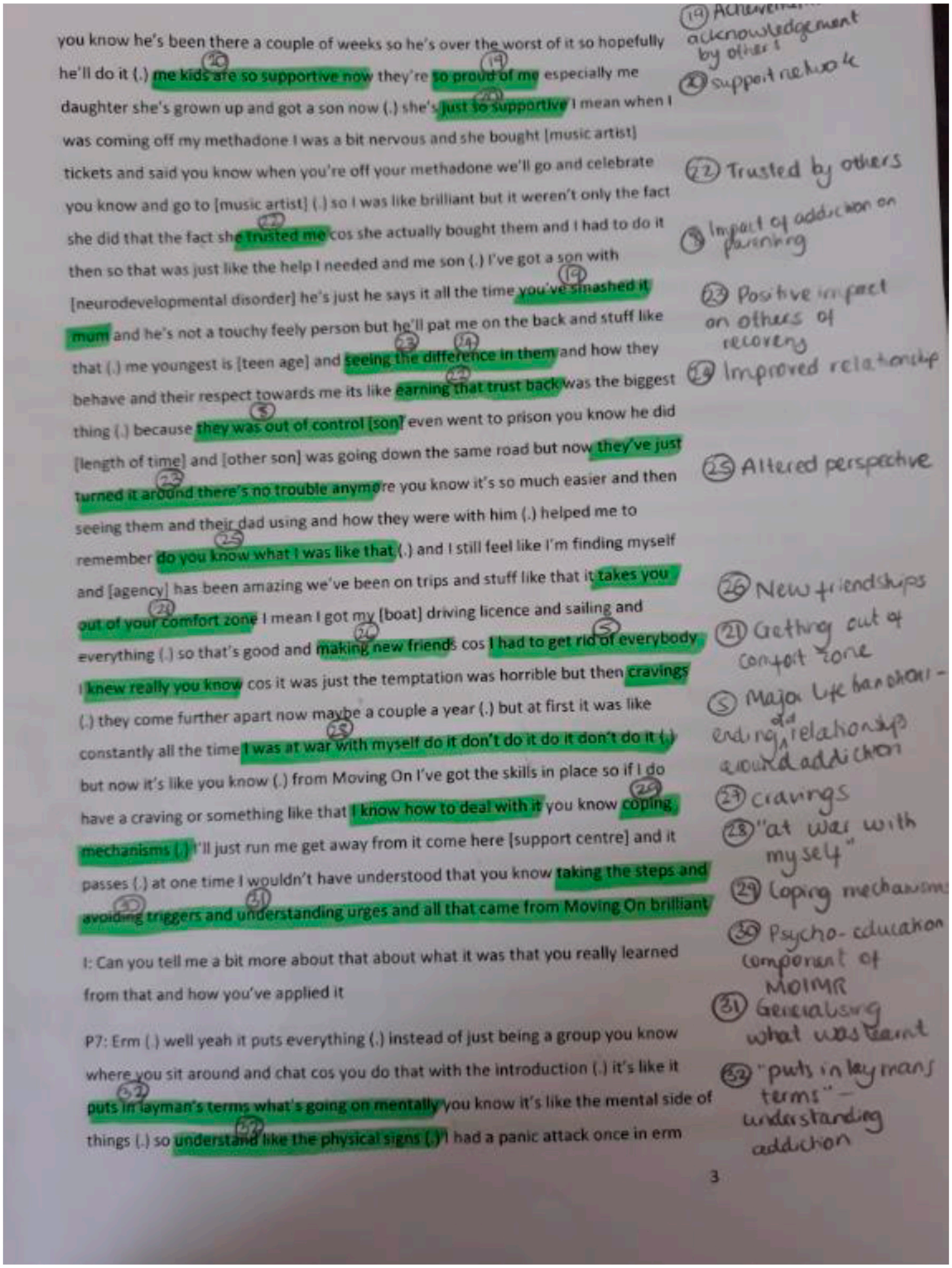

you know he's been there a couple of weeks so he's over the worst of it so hopefully he'll do it (.) me kids are so supportive now they're so proud of me especially me daughter she's grown up and got a son now (.) she's just so supportive I mean when I was coming off my methadone I was a bit nervous and she bought [music artist] tickets and said you know when you're off your methadone we'll go and celebrate you know and go to [music artist] (.) so I was like brilliant but it weren't only the fact she did that the fact she trusted me cos she actually bought them and I had to do it then so that was just like the help I needed and me son (.) I've got a son with [neurodevelopmental disorder] he's just he says it all the time you've smashed it mum and he's not a touchy feely person but he'll pat me on the back and stuff like that (.) me youngest is [teen age] and seeing the difference in them and how they behave and their respect towards me its like earning that trust back was the biggest thing (.) because they was out of control [son] even went to prison you know he did [length of time] and [other son] was going down the same road but now they've just turned it around there's no trouble anymore you know it's so much easier and then seeing them and their dad using and how they were with him (.) helped me to remember do you know what I was like that (.) and I still feel like I'm finding myself and [agency] has been amazing we've been on trips and stuff like that it takes you out of your comfort zone I mean I got my [boat] driving licence and sailing and everything (.) so that's good and making new friends cos I had to get rid of everybody I knew really you know cos it was just the temptation was horrible but then cravings (.) they come further apart now maybe a couple a year (.) but at first it was like constantly all the time I was at war with myself do it don't do it do it don't do it (.) but now it's like you know (.) from Moving On I've got the skills in place so if I do have a craving or something like that I know how to deal with it you know coping mechanisms (.) I'll just run me get away from it come here [support centre] and it passes (.) at one time I wouldn't have understood that you know taking the steps and avoiding triggers and understanding urges and all that came from Moving On brilliant

I: Can you tell me a bit more about that about what it was that you really learned from that and how you've applied it

P7: Erm (.) well yeah it puts everything (.) instead of just being a group you know where you sit around and chat cos you do that with the introduction (.) it's like it puts in layman's terms what's going on mentally you know it's like the mental side of things (.) so understand like the physical signs (.) I had a panic attack once in erm

[town] bus station and there was no reason I should have a [illegible] heart started beating and it put me on the floor I couldn't breathe and I understood what helped me come out the panic attack cos I was on my own was oh I'm having a physical craving I know what I'm doing you know (.) and understanding that instead of just panicking and going to score you know (.) it helped that it's like that fight or flight you know even on the session when they talk about winning a erm safari trip to Africa and with the lion (.) you know so I understand that I'm having a panic attack and I'm just craving you know so my mind is tricking me kind of thing so it helped me bring it out of it you know to understand what was going on and to take it instead of getting all these feelings all these emotions and not knowing like I had done before (.) to be able to break it down and understand and how to deal with cravings as well like the salesman (.) you know let him and say it's ok but no you know (.) so instead of being at war constantly with yourself it really did it changed my life you know it was really good (.) but I think it took me a couple of times I think at first once I'd done it I was still coming out of it and I think it was after I'd done all 3 that I started to come to understanding and put it in to practice you know I mean I did it before but it was just like now I know (.) it took a couple of times I think yeah

I: So you alluded before to like the salesman knocking on your door (.) letting him in (.) what's that kind of meant to you

P7: Well before I was just like no no no don't don't don't (.) and you're mentally at war with yourself but my understanding ok (.) I'm just craving (.) accepting that I'm craving and then saying no thank you (.) you know I'm not doing it today (.) it's like a weight of the world off your shoulders (.) it's not like that good angel and little devil on your shoulders that do it don't do it (.) you just accept it and it just helps you mentally (.) cos my mental health weren't great after all that you know and I was going through a hard time and a break up with [ex-partner's name] so yeah it just really really did help so yeah definitely

I: And is there anything else that you've really picked up from the group that's really stuck really stayed with you

P7: Erm (..) I think unintentionally I think I put it in erm words but me own words you know like helping other people like me being a volunteer and not only me but like I said [partner]'s in rehab so and he's having a really hard time well at the beginning he was and I found myself using Moving On you know erm to try but breaking it

(31) Generalising

(33) Understanding allows to make [illegible] for uncomfortable sensations

(34) "my mind is tricking me" → defusion → distance from thoughts

(35) Making space for discomfort ↓ opposite to

(36) Overwhelmed by emotions / desire to avoid

(37) instead of 'being at war with self'

(38) Took doing the group x3 to fully apply + understand

(39) Accepting → relief

(40) Not being ruled by thoughts/cravings → distance

(41) Relief

(42) Using own learning to help others

Category generation:

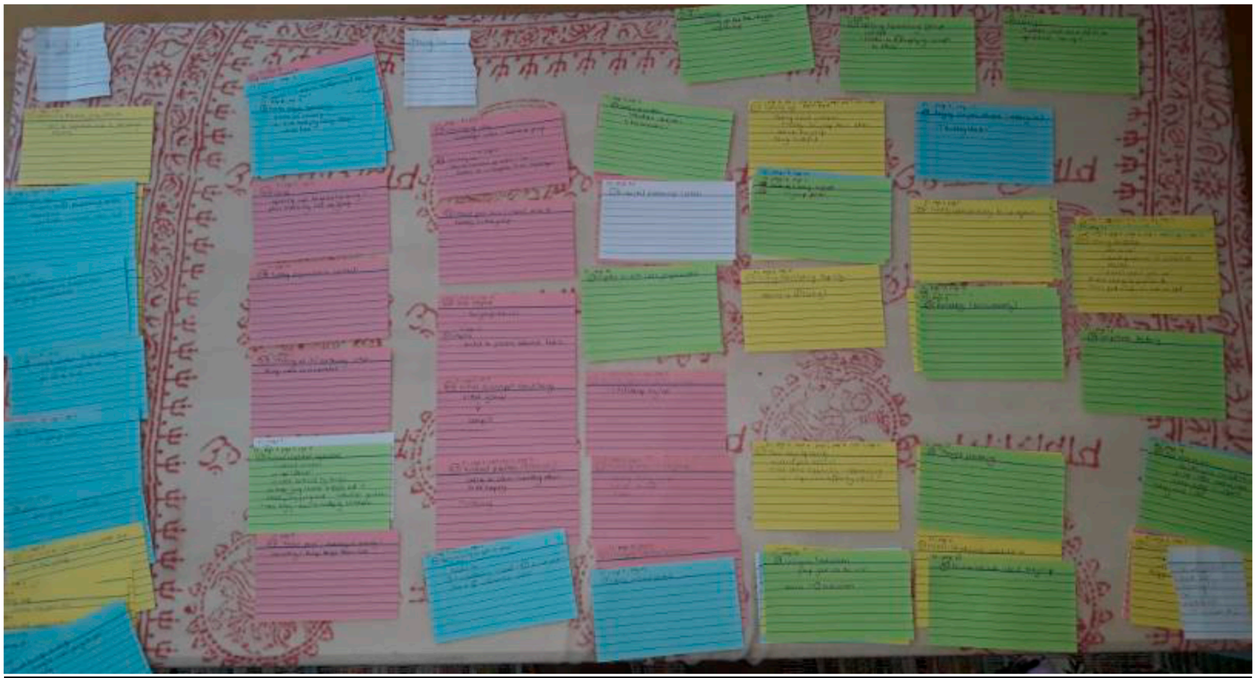

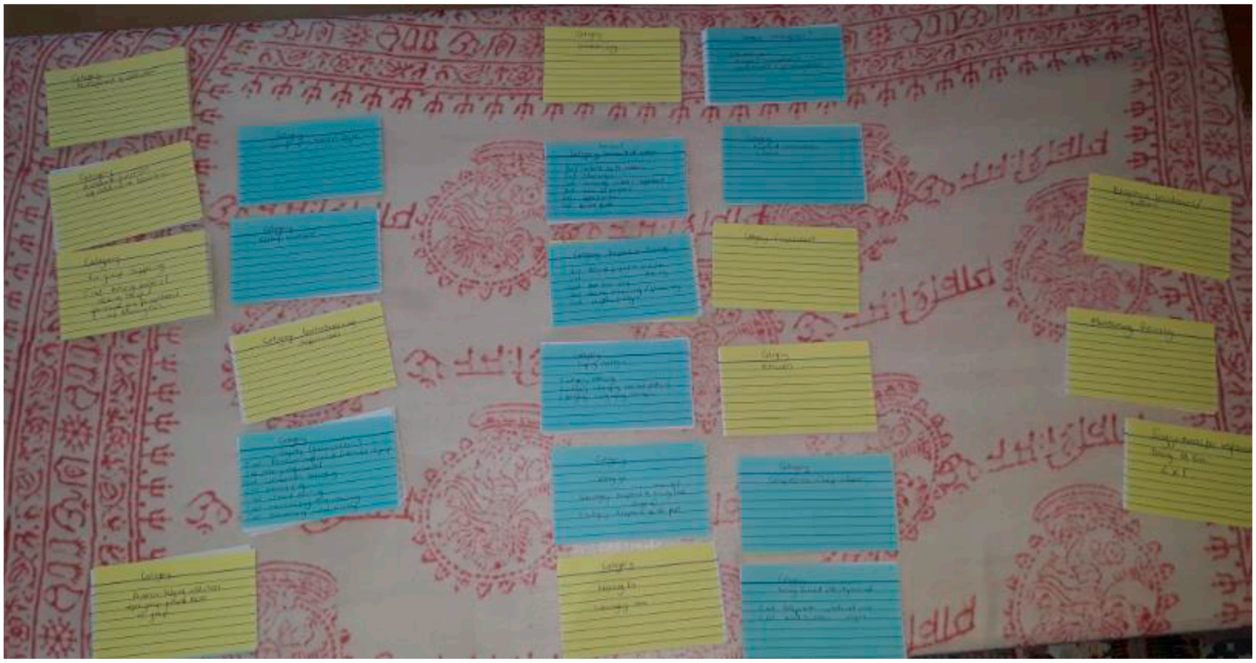

Memo excerpt:

14/02/2020: memoing on p9 transcript

P9 talks about the difficulty of some of the processes – acceptance of addiction, refers to it as a "big hurdle", and that movement from shame/ wanting to hide addiction to acceptance of it (page 10)…. This emphases the difficulty of applying these constructs. He also discusses earlier in the transcript how it took him completing the group three times before he began to feel able to accept the addiction and what others thought of him.

P9 talks about the stigma session, shifting from a life of an addict and the identity of an addict, but then also discusses the fear of who he is without drugs, and how the content of that session seemed to have given him the "tools" (in his words) to build a new life. This, combined with the positive relationships formed in the group, supported the process of developing a new identity as someone in recovery.

15/02/2020: memoing on p10 transcript

P10 discusses the role of the group community, this makes me think about the attachment/ sense of family/ belonging that was clearly missing in P9's life that this provided.

The prioritising process that needs to happen could be seen like a weighing scale – investing more in to recovery so that is the heavier end, so that there is so much that could be lost by going back to alcohol/ drugs; P10 talks about the pros and cons outweighing each other (page 5). This is something I am seeing in all the transcripts, this sense of life without drugs becoming more rewarding and far outweighing a life with drugs.

Another key process seen here is learning to accept difficult life experiences, make space for difficult emotions – "life's not perfect" as P10 talks about.

PAGE 9 about learning that blocking out (experiential avoidance) leads to further and maintained difficulty

A note on language: the accessible language used (rather than ACT language) seems to mean that participants have been able to apply it to their own lives, P10 uses phrases that are taken directly from the group, as do so many other participants.

Diagrams of Model Development (Versions 1-3):

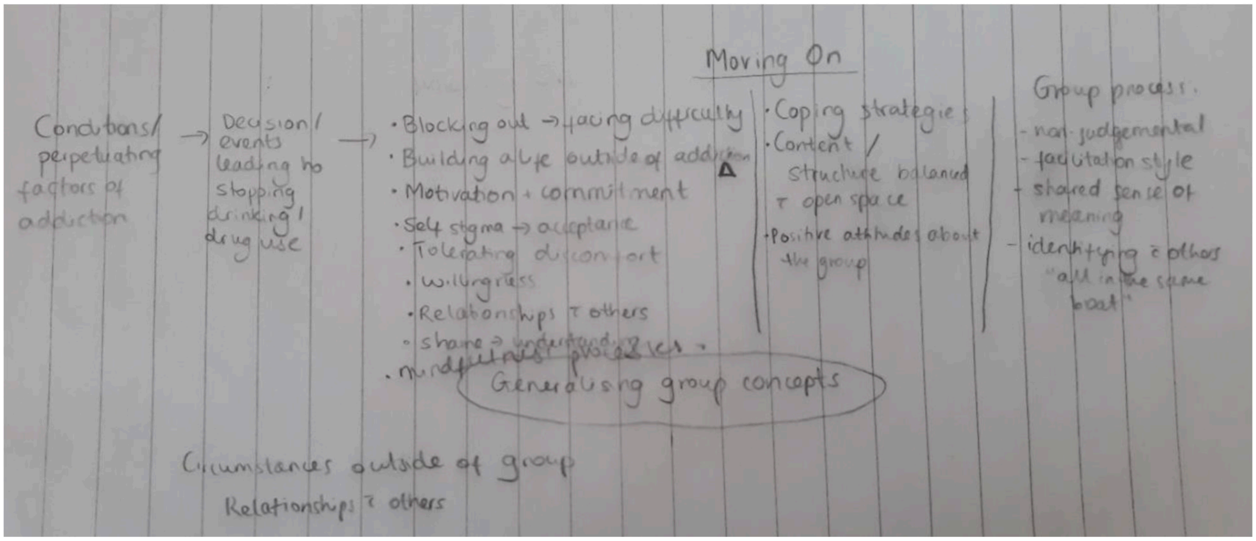

z

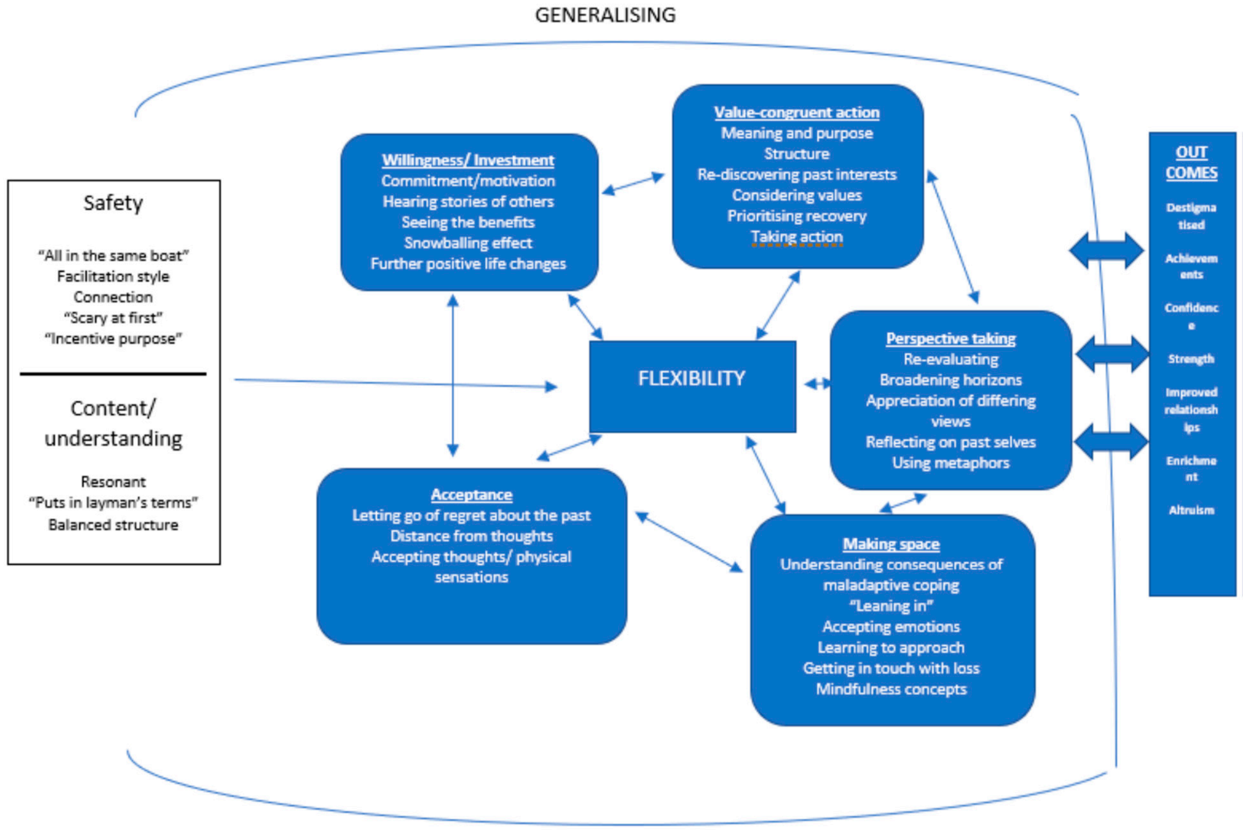

Integrating = outcomes.

Suffering → Safety / Understanding → Investment / Action → ~~Sense of self~~ Making space / Acceptance → Sense of self

Taking time

Core category: more value / reward in recovery / feedback / reinforcements?
